# Left ventricular assist device in pregnancy: case report of a near-term delivery with multidisciplinary support

**DOI:** 10.1093/ehjcr/ytag513

**Published:** 2026-07-10

**Authors:** Megan E Bunnell, Tianjiao Ren, Tyler J Guidugli, Bushra W Taha, Katherine E Economy, Michael M Givertz

**Affiliations:** Division of Maternal–Fetal Medicine, Brigham and Women’s Hospital, Harvard Medical School, 75 Francis Street, Boston, MA 02115, USA; Division of Cardiovascular Medicine, Brigham and Women’s Hospital, Harvard Medical School, 75 Francis Street, Boston, MA 02115, USA; Department of Anesthesiology, Perioperative and Pain Medicine, Brigham and Women’s Hospital, Harvard Medical School, 75 Francis Street, Boston, MA 02115, USA; Department of Anesthesiology, Perioperative and Pain Medicine, Brigham and Women’s Hospital, Harvard Medical School, 75 Francis Street, Boston, MA 02115, USA; Division of Maternal–Fetal Medicine, Brigham and Women’s Hospital, Harvard Medical School, 75 Francis Street, Boston, MA 02115, USA; Division of Cardiovascular Medicine, Brigham and Women’s Hospital, Harvard Medical School, 75 Francis Street, Boston, MA 02115, USA

**Keywords:** Heart failure, Pregnancy, Peripartum cardiomyopathy, Left ventricular assist device, Case report

## Abstract

**Background:**

Left ventricular assist devices (LVADs) are utilized to treat end-stage heart failure (HF) refractory to pharmacologic and device-based therapies. LVAD use is considered a relative contraindication to pregnancy due to concerns for increased maternal and foetal risk. Nonetheless, there is an increasing prevalence of LVAD use in pregnant patients with advanced HF. Data have been limited to case reports without guidelines for pregnancy management, and there are no reports of delivery beyond the 34th week of pregnancy.

**Case:**

We present a case of a pregnant person with an LVAD who underwent an uncomplicated near-term induction with an unscheduled caesarean delivery without cardiac decompensation or hypertensive disorders. She received multidisciplinary care throughout her antenatal, intrapartum, and postpartum course.

**Conclusion:**

In LVAD patients with stable haemodynamics, managed by a multidisciplinary team in an appropriately resourced hospital, there may not be a need for planned preterm delivery.

Learning points

- Pregnancies in LVAD users are increasing with improvement in safety and outcome data.
- Risks are multifactorial but can be minimized with a multidisciplinary team approach.
- In stable LVAD patients, there may not be a need for planned preterm delivery.

## Introduction

Mechanical circulatory support (MCS) is utilized to treat advanced heart failure (HF) refractory to medical therapies. Pregnancy in patients requiring left ventricular assist device (LVAD) support is classified as modified World Health Organization Class IV, reflecting an extremely high risk of maternal morbidity and mortality, even in clinically stable individuals without recurrent HF. The presence of an LVAD is considered a relative contraindication to pregnancy due to concerns for maternal and foetal risk.^1^ There is an increased incidence of LVADs in pregnant patients with advanced HF.^1,2^

There are limited reports detailing the outcomes of pregnancies in the setting of durable MCS, summarized in this manuscript. We present a case of pregnancy in a patient with an LVAD resulting in a 36-week induction and urgent caesarean delivery managed by a multidisciplinary team on labour and delivery without maternal or neonatal morbidity.

## Summary figure

**Figure:**
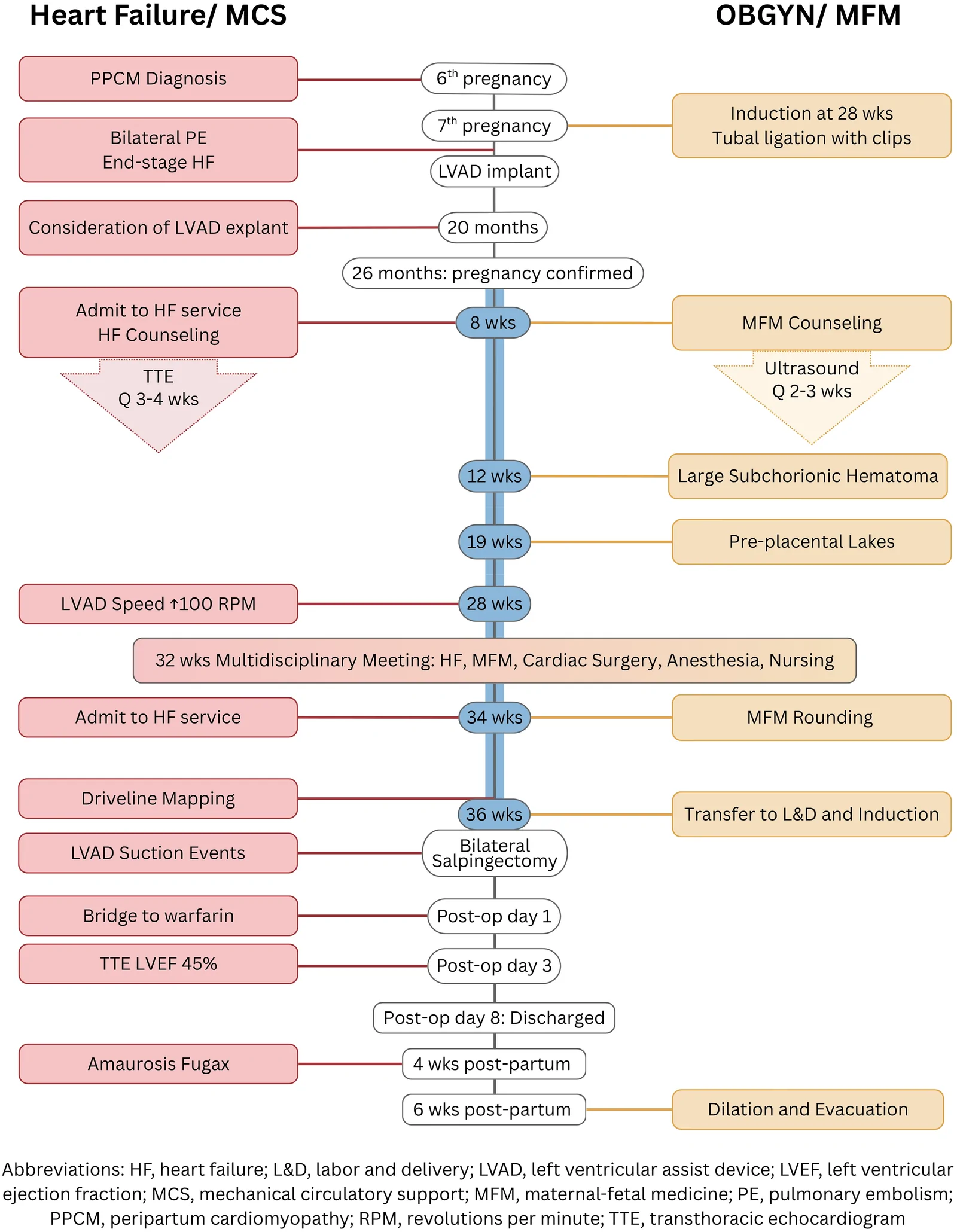


## Case

The patient is a White, non-Hispanic 38-year-old G8P6107 (eight pregnancies, six prior term deliveries, one preterm delivery, no miscarriages, and seven living children) female with a history of five uncomplicated term vaginal deliveries. After her sixth-term delivery, she developed acute systolic HF and was diagnosed with peripartum cardiomyopathy in the setting of polysubstance use (nicotine, methamphetamine, and tetrahydrocannabinol). Echocardiogram showed left ventricular (LV) end-diastolic dimension 72 mm, left ventricular ejection fraction (LVEF) 20%, and moderate right ventricular (RV) dysfunction.

She was started on medical therapy for HF, which she self-discontinued after 6 months as she was feeling better. She had an unplanned pregnancy the following year. That (seventh) pregnancy was complicated by recurrent HF necessitating delivery at 28 weeks, after which she elected to undergo bilateral tubal ligation via the clip method. Six weeks postpartum, she presented to an outside hospital with chest pain and shortness of breath and was found to have bilateral pulmonary emboli on chest CT angiography.

She was started on heparin and transitioned to apixaban. Echocardiogram showed severe LV dilation with estimated LVEF 20%, dilated RV with low normal function, severe mitral regurgitation, and moderate tricuspid regurgitation with evidence of elevated right and left atrial pressures and moderate pulmonary hypertension. A right heart catheterization demonstrated a low output state with cardiac index 1.0 L/min/m^2^, pulmonary capillary wedge pressure 26 mmHg, and systemic vascular resistance 3000 dynes/s/cm^−5^. She was treated with dobutamine 3 mcg/kg/min, diuresed with IV furosemide, and continued on home midodrine 2.5 mg TID for additional BP support. Five days after admission, dobutamine was discontinued, and she was started on sacubitril-valsartan. However, she developed symptomatic hypotension, which prompted re-initiation of dobutamine and transfer to our centre.

Upon transfer, she was stabilized on medical therapy including dobutamine. She was discharged home on continuous IV inotropic therapy with a plan to assess for adherence to medical care and monitor for substance use abstinence while considering evaluation for advanced HF therapies. Over the next few months, her clinical status worsened despite home inotropic support, and she was readmitted 3 months later. Her NT-proBNP level had up-trended from 3122 pg/mL to 7949 pg/mL, and right heart catheterization revealed RA 11 mmHg, PA 55/35/42 mmHg, PCWP 29 mmHg, cardiac index 2.1 L/min/m^2^, and pulmonary vascular resistance 480 dynes/s/cm^−5^. Having maintained abstinence from methamphetamine use, she underwent HeartMate 3 (HM3; Abbott Cardiovascular, Plymouth, MN) LVAD placement as a bridge to transplant candidacy given active marijuana use and high burden of allosensitization. She was followed closely by cardiology with no major adjustments in her initial LVAD settings. She was maintained on sacubitril-valsartan, metoprolol, spironolactone, empagliflozin, warfarin, and aspirin.

With LVAD and medical therapy, she was satisfied with her quality of life and deferred heart transplantation. LVEF at 1 year post-LVAD was 45%–50% and the possibility of device explantation in the coming year was discussed. During this year (2 years after initial LVAD placement), the patient called her cardiology office with feelings of lethargy and nausea, missed menses and a positive home pregnancy test. On ultrasound, she was found to have an 8-week-sized intrauterine pregnancy and was admitted to the HF service for coordination of care, counselling, and multidisciplinary management.

Counselling by maternal–foetal medicine (MFM) and HF teams included risk of first trimester high dose warfarin exposure, risk of transitioning off guideline-directed medical therapy (GDMT) during pregnancy, potential for recurrent cardiomyopathy, the increased hypercoagulable state of pregnancy, and complications from haemodynamic changes in pregnancy including increased plasma volume, fluid shifts postpartum, and increased cardiac demand. After multidisciplinary team discussions and patient-centred decision-making, she elected to continue with the pregnancy.

She transitioned from warfarin to enoxaparin with a peak anti-Xa target of 0.8 to 1.2 IU/mL.^1^ She discontinued GDMT (sacubitril-valsartan, spironolactone, and empagliflozin) and initiated metoprolol, hydralazine, and aspirin owing to their improved safety profile in pregnancy. First trimester LVAD parameters were unchanged from pre-pregnancy settings, and she was discharged to outpatient management without activity restrictions. Her NT-proBNP level remained stable, ranging from <36 to 149 pg/mL. She was followed by psychiatry and counselled regarding the importance of cannabis cessation in pregnancy, as prenatal cannabis exposure has been associated with adverse perinatal outcomes, including lower birth weight, foetal growth restriction, and a possible increased risk of preterm birth.^3^

Ultrasound at 12 weeks’ gestation showed a normal nuchal translucency and low-risk aneuploidy screening with a large subchorionic haematoma. Given maternal risks of thrombosis and otherwise typically developing pregnancy without associated bleeding, no changes to anticoagulation were made. She continued enoxaparin and aspirin, the latter of which was indicated for obstetric prevention of preeclampsia. She was followed weekly with ultrasounds showing resolution of the subchorionic haematoma and normal foetal development. Her 19-week anatomy ultrasound was overall unremarkable.

**Figure 1:**
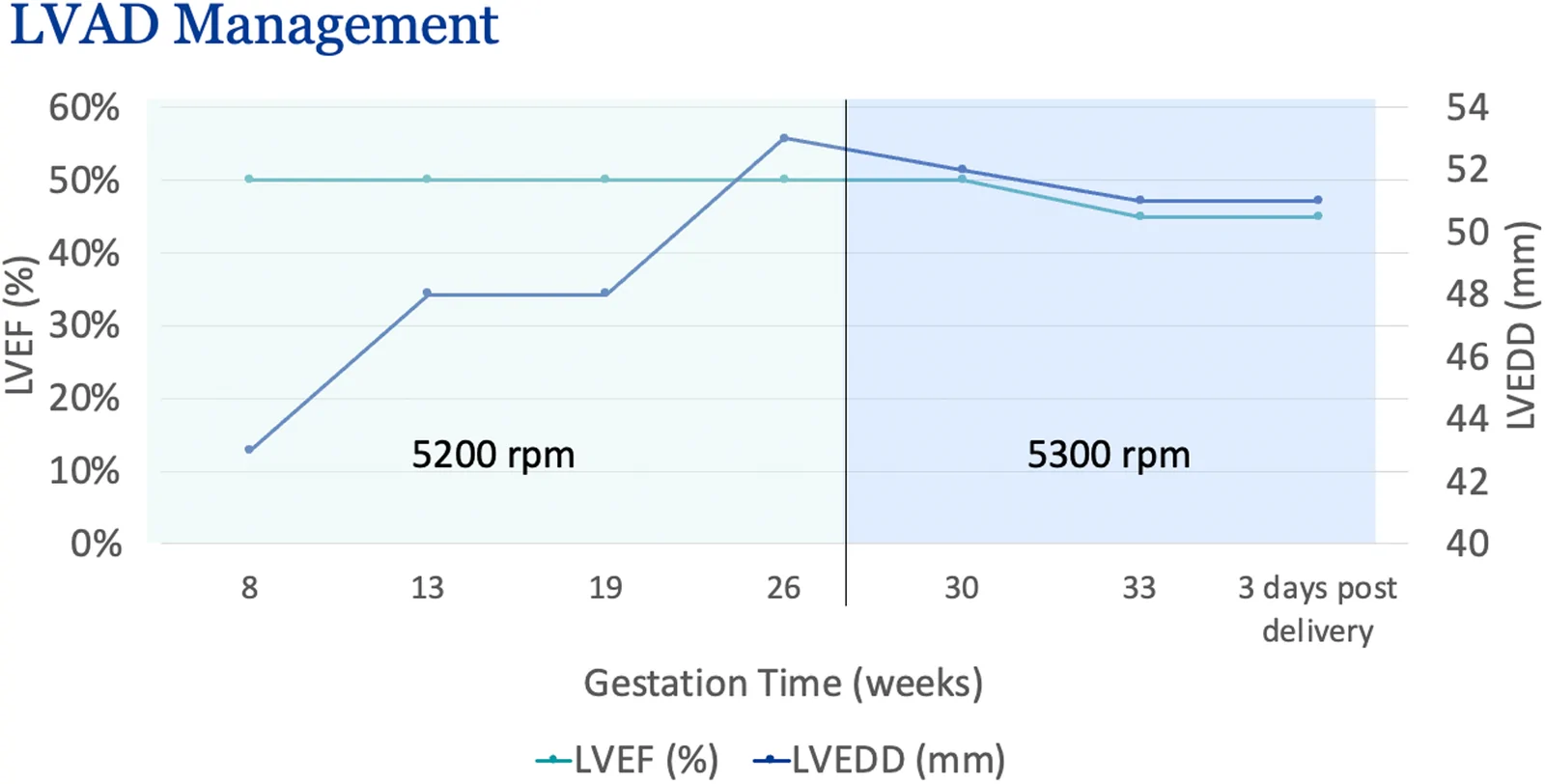
Measurements of left ventricular ejection fraction (LVEF) and left ventricular end-diastolic dimension (LVEDD) over time during the pregnancy with change in LVAD speed.

Cardiac concerns included the risk of recurrent HF and hypertensive disorders of pregnancy. She continued weekly visits, during which she maintained stable New York Heart Association functional Class I–II symptoms and consistently normal blood pressures. She had biweekly transthoracic echocardiograms to assess cardiac and LVAD function. A representative echocardiogram at 33 weeks gestation is demonstrated, showing mildly reduced LV systolic function, dilated right ventricle, and reduced RV systolic function without aortic regurgitation (see Clip *S1*). Over the course of pregnancy, LVAD speed was increased once by 100 RPM at 28 weeks. Her LVEF remained stable at 45%–50% throughout pregnancy (*Figure 1*).

At 32 weeks gestation, a multidisciplinary meeting was held for delivery planning with representatives from cardiac surgery, HF cardiology, MFM, nursing, and obstetric anaesthesiology. This patient was admitted at 34 weeks to the HF service for care coordination with a plan for delivery at 36 weeks. Her cardiac status was stable at the time of admission with NT-proBNP level of 60 pg/mL. This gestational age was selected to minimize the potential of late third-trimester hypertensive complications and unscheduled delivery and to minimize the risks of prematurity. At the time, there were no reports of LVAD-assisted pregnancies continuing beyond the 34th week of pregnancy.

The day preceding delivery, she underwent LVAD driveline mapping via ultrasound to mark the surgical course on the skin in case of an unplanned caesarean delivery. She was transferred to the labour and delivery floor on the day of the scheduled induction. This labour and delivery floor functions as an intensive care unit (ICU) and is equipped for arterial line monitoring and telemetry with dedicated obstetric anaesthesiologists. An MCS nurse remained at bedside throughout the duration of care to monitor for changes in LVAD parameters. She was transitioned to a heparin drip, which was held 4 h prior to epidural placement. An arterial line was placed for vital sign monitoring. Vertex presentation was confirmed via ultrasound, and a balloon was placed for cervical ripening. After removal of the balloon, an epidural was placed, and she was started on oxytocin for augmentation of contractions. Six hours following artificial rupture of membranes, the foetus was noted to have changed to breech presentation with a concerning foetal heart rate tracing unresponsive to typical interventions (e.g. maternal repositioning). The patient was transferred to the labour and delivery operating room for urgent primary caesarean delivery and bilateral salpingectomy (for permanent sterilization purposes given prior clip failure). This was discussed with the patient during prenatal care as an option if caesarean delivery was otherwise indicated.

Surgery was uncomplicated with an estimated blood loss of 450 cc. Her epidural catheter was initially dosed with 5 mL 2% lidocaine with 1:200 000 epinephrine followed by 15 mL of 3% chloroprocaine. Supplemental IV fentanyl, midazolam, and inhaled nitrous oxide were administered until adequate surgical anaesthesia was achieved. Haemodynamic stability was maintained with IV norepinephrine, and albumin and crystalloid solutions were utilized for fluid resuscitation. Vasopressor support from hysterotomy closure until the immediate postoperative period was achieved with low-dose (5 mcg/min) norepinephrine.

A male baby was delivered from the breech presentation weighing 2.7 kg with Apgar scores of 8 and 9 at 1 and 5 min, respectively. He was transferred to the neonatal ICU due to the need for respiratory support via continuous positive airway pressure and had a total length of stay of 14 days (38 weeks corrected gestational age). After placental delivery and during hysterotomy closure, the patient experienced LVAD suction events that were treated with additional albumin resuscitation and escalating doses of norepinephrine, which were able to be weaned in the immediate post-operative period.

The patient was transferred to the cardiac ICU for recovery. She was restarted on a heparin drip on postoperative Day 1 and bridged to warfarin with an INR goal of 2–3. She initiated breastfeeding. Echocardiogram on postpartum Day 3 showed LVEF 45%, and no changes were made to her LVAD settings. She was discharged on postoperative Day 8 on metoprolol, warfarin and aspirin. Placenta pathology revealed a small for gestational age placenta, low-grade foetal vascular malperfusion, and mild decidual arteriopathy with subchorionic haemorrhage encompassing 10% of placental disc. She was seen in clinic on postoperative Day 14 and was feeling well without dyspnoea, palpitations, or oedema. She was no longer breastfeeding and was started on sacubitril-valsartan and spironolactone.

At 4 weeks post-partum, she presented to the emergency department with an episode of vision loss in the upper half of her right eye. This resolved spontaneously and recurred 30 min later. Head and neck CT angiography was negative, and she was started on heparin for presumed amaurosis fugax due to an embolic event. INR was noted to be subtherapeutic at 1.7. Ophthalmology exam and follow-up neurologic exams were unremarkable. Her warfarin dose was increased, and she was restarted on a 14-day enoxaparin bridge. She was seen 10 days later with increased vaginal bleeding and started on a progesterone taper.

Pelvic ultrasound revealed a 2-cm area of possible retained products of conception. Under general anaesthesia, she underwent diagnostic hysteroscopy and dilation and evacuation. Pathology revealed evidence of retained products (degenerated chorionic villi and placental implantation site). A levonorgestrel intrauterine device was placed for control of menses in the setting of lifelong anticoagulation. She recovered well and reported no further bleeding two weeks following this procedure. At time of manuscript submission (12 months postpartum), she and her baby were well with no unanticipated medical needs.

## Discussion

**Figure 2:**
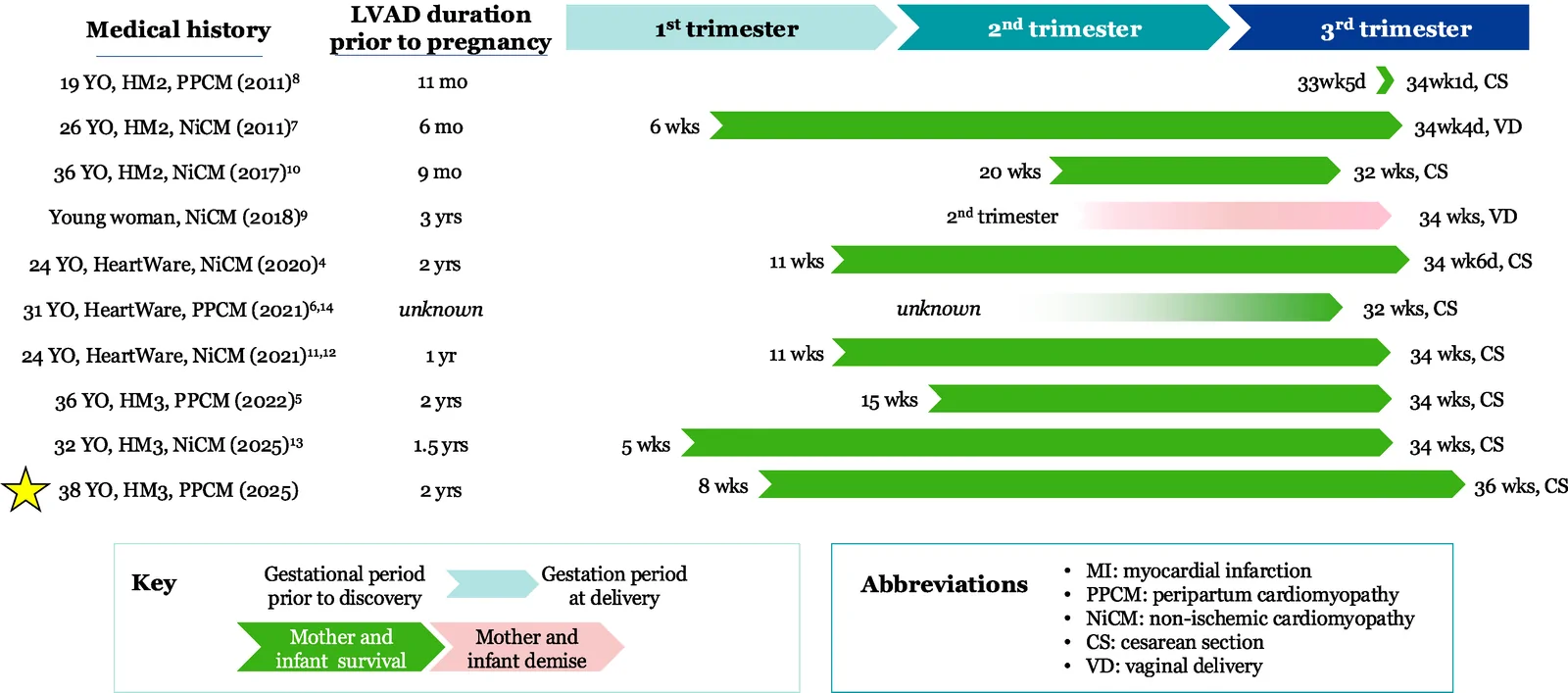
Representation of all current available case reports of LVAD-supported pregnancies including length of prenatal care, delivery timing and maternal/infant outcome including this case.

There have been eight viable pregnancies supported by LVAD therapy.^4,5,6^ Most of these successful cases involved: (1) early initiation of a multidisciplinary team including obstetricians and cardiologists, (2) heparin initiation as a transition from warfarin at the time of pregnancy detection, (3) mild increase in LVAD speed to support pregnancy-related increased cardiac output in the second and third trimester, and (4) successful delivery between 28 and 34 weeks with maternal postpartum complications including anaemia and acquired von Willebrand syndrome (*Figure 2*).^7^

Of the eight pregnancies previously reported, three were supported with the HeartWare HVAD (Medtronic), three with HeartMate II LVAD (Abbott), and two with HM3; one report did not include the device used.^8–12^ The most recent case report was the second involving HM3 support, the same LVAD implanted in our patient.^13^ In both cases, enoxaparin was dosed to target a peak anti-Xa level of 0.8–1.2 IU/mL.^13^ While neither was associated with thrombotic or bleeding complications, the limited sample size is insufficient to make definitive recommendations regarding anticoagulation in this setting.

**Figure 3:**
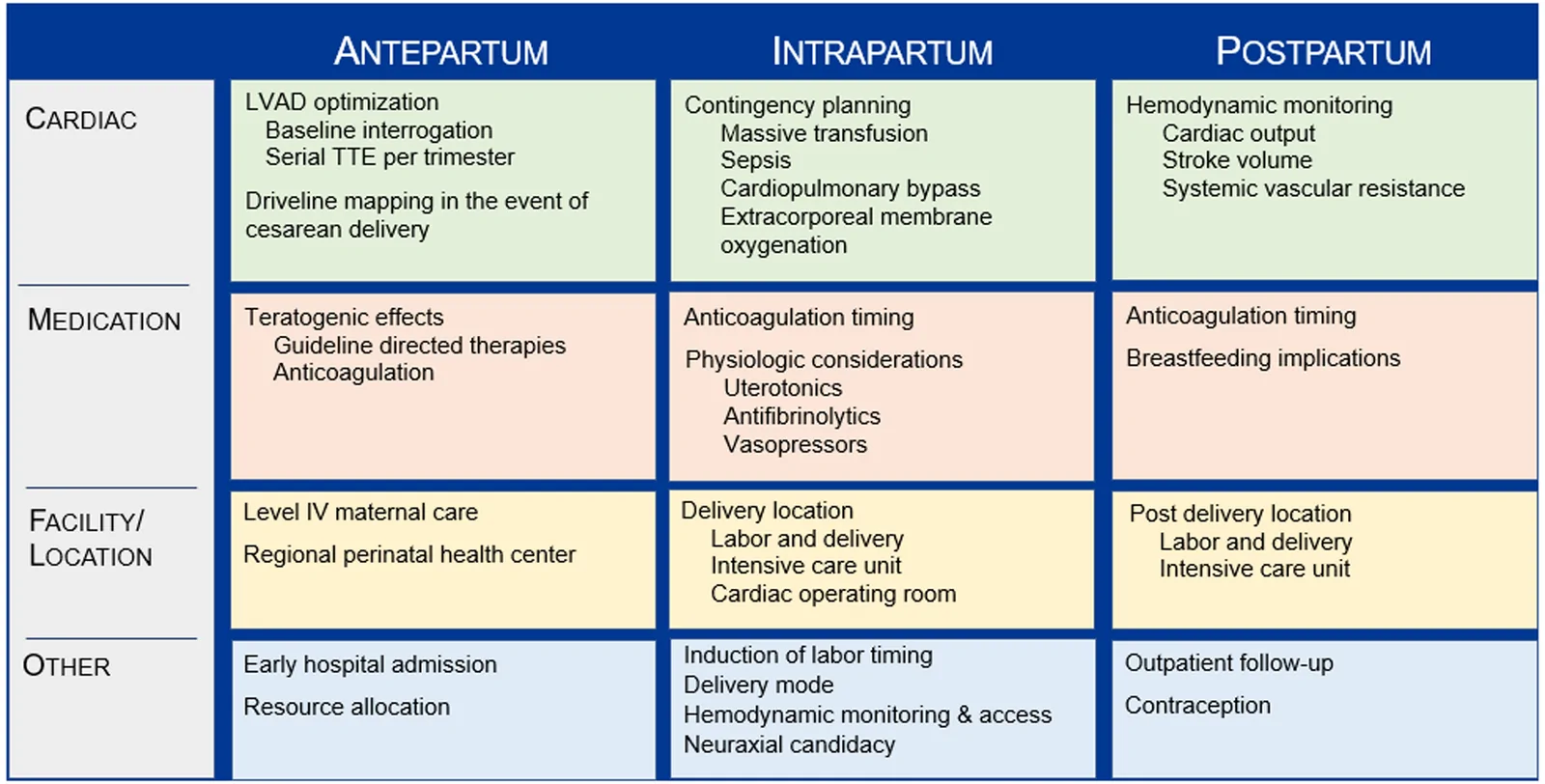
Summary of key antepartum, intrapartum and postpartum considerations in management of LVAD in pregnancy.

There are unique considerations for the antepartum, intrapartum and postpartum management of patients supported with an LVAD (*Figure 3*). Antepartum, each trimester presents different challenges in patients with durable MCS. As pregnancy progresses, physiological changes such as increased plasma volume put weakened myocardium at risk for volume overload requiring an increase in LVAD speed to maintain adequate cardiac output. This in turn can increase the risk of RV failure. Risks associated with pregnancy in the setting of LVAD support are multifactorial but can be minimized with a multidisciplinary team approach. In the case of the patient presented here, all providers involved in her care met as a group each trimester and just prior to delivery to discuss anticipated issues, potential complications, and contingency planning should, as ultimately occurred, a caesarean delivery be warranted in an urgent or emergent way. These meetings included input from provider teams, including cardiology, MFM, obstetric and cardiac anaesthesiology, infectious disease, psychiatry, and haematology, and additionally included perspectives from systems-based leadership teams, including nursing on both cardiology and obstetric floors. This type of longitudinal meeting allowed teams to consider issues requiring multi-specialty input, for example, how to coordinate anticoagulation timing with induction planning and antibiotic administration.

As was discussed amongst all teams, in LVAD users during pregnancy, thromboembolism risk is increased due to the hypercoagulability of pregnancy and additional risks associated with device surfaces and blood stasis. As a result, anticoagulation is required throughout pregnancy, and this can increase risk of bleeding, especially from mucosal surfaces, and impact the eligibility for neuraxial anaesthesia. Driveline infections are common in LVAD patients with estimated rates ranging from 15% to 30% per year of LVAD use, though no specific pregnancy-associated data exist.^14^ Antibiotic choice is more limited in pregnancy, and the consequences of systemic infection in pregnancy can result in preterm birth, maternal sepsis, and/or foetal demise.^5,15,16^

Delivery planning for patients with LVAD in pregnancy also requires multidisciplinary, patient-focused care. Planning in our case involved input from multiple clinical teams, and cardiac ICU and labour floor nursing. Her labour was able to be managed entirely on the obstetric floor, with an urgent caesarean section performed on the labour and delivery unit under neuraxial anaesthesia. The possibility of this outcome was discussed in advance, as mentioned, including the determination that it was safest for her to labour and be delivered in the obstetric area of our hospital rather than on a cardiac-specific floor. This provision of care was made possible with support from all teams and careful antepartum and intrapartum considerations of obstetric medications and interventions on device physiology, judicious timing of anticoagulation, and contingency planning in the event of haemodynamic collapse.

Postpartum considerations include the management of rapid changes in physiological circulation following placental delivery and in the event of common obstetric complications. The case presented here demonstrates that this can be safely achieved under neuraxial anaesthesia in an urgent setting with appropriate haemodynamic support.^2,5^ Resumption of anticoagulation requires consideration of many factors, including mode of delivery, breastfeeding goals, and potential for delayed postpartum bleeding. Finally, the decision regarding the timing of delivery should be carefully considered as part of multidisciplinary management.

As with any complex delivery, the neonatal risks associated with prematurity need to be balanced with the maternal risks of ongoing pregnancy, including the escalating haemodynamic stresses of the end of the third trimester and the usual pregnancy-related risks of hypertension, preeclampsia and thrombocytopenia. While those risks are more significant in individuals with underlying cardiac compromise, there are many complex maternal conditions that are managed to 37 weeks. In the limited available literature, including this case, management to early term may have been appropriate, with careful consideration of inpatient vs. intensive outpatient surveillance.

No prior cases of LVAD in pregnancy discuss specifically the importance of postpartum contraception and contraceptive counselling. This patient underwent tubal ligation in a prior pregnancy using Filshie clips, a common method of sterilization with a 10-year cumulative probability of failure of 4.1–23.3/1000 procedures performed.^17^ While no contraceptive method short of hysterectomy is perfect, the importance of a safe and adherable contraceptive plan should be discussed and documented with every patient pursuing LVAD therapy to prevent unintended pregnancy. Furthermore, in LVAD users who are pregnant, the potential for opportunistic permanent sterilization in the form of bilateral salpingectomy in the event of caesarean delivery should be discussed.

There remain many gaps in knowledge about how to best manage the pregnant LVAD patient. As we gather more information and experience, many of these risks may be better delineated and minimized, increasing the safety of pregnancy in the context of durable MCS. Furthermore, in patients with stable haemodynamics, managed by a multidisciplinary team in an appropriate resourced hospital, there may not be a need for a planned preterm delivery.

## Supplementary Material

ytag513_Supplementary_Data

## Data Availability

The data presented here describe a clinical case; all available data are included within the manuscript. Additional details can be provided upon request.

## Lead author biography

Megan Bunnell, MD, MS, is a maternal–foetal medicine and genetics fellow at the Brigham and Women’s, Massachusetts General, Boston Children’s combined fellowship programme. Her research interests include prenatal care, foetal genetics, and advanced foetal imaging. She has worked on a number of cross-collaborative efforts with departments across the hospital with the goal of improving prenatal care for complex patients and hopes to continue to practice in this area.

## Notes

**Consent:** Written informed consent was obtained from the patient for publication of this case report and any accompanying images. The authors confirm that all efforts have been made to protect patient privacy and anonymity. This work complies with the Committee on Publication Ethics guidelines.

## Case Presentation

This case was presented at the Society for Obstetric Anesthesia and Perinatology (Portland OR) (4/2025) as a poster titled: Emergent Intrapartum Cesarean Delivery in a Grand Multiparous Parturient Supported by a Left Ventricular Assist Device (LVAD)
