# Supplementary material for: Left ventricular assist device in pregnancy: case report of a near-term delivery with multidisciplinary support
Source: Eur Heart J Case Rep. 2026 Jul 10;10(8):ytag513. doi: 10.1093/ehjcr/ytag513 (PMC13594975; doi:10.1093/ehjcr/ytag513)
Supplement: Supplementary file 1 [file ytag513_Supplementary_Data.zip › video_Legends.docx]

**Clip Legends**

**Clip 1:** 33 week echocardiogram
